# Investigating the racial gap in prostate cancer screening with prostate-specific antigen among younger men from 2012 to 2020

**DOI:** 10.1093/jncics/pkad003

**Published:** 2023-01-27

**Authors:** Zhiyu Qian, Khalid Al Khatib, Xi Chen, Sanvi Belani, Muhieddine Labban, Stuart Lipsitz, Alexander P Cole, Hari S Iyer, Quoc-Dien Trinh

**Affiliations:** Division of Urological Surgery, Brigham and Women’s Hospital, Harvard Medical School, Boston, MA, USA; Center for Surgery and Public Health, Brigham and Women’s Hospital, Harvard Medical School, Boston, MA, USA; Division of Urological Surgery, Brigham and Women’s Hospital, Harvard Medical School, Boston, MA, USA; Center for Surgery and Public Health, Brigham and Women’s Hospital, Harvard Medical School, Boston, MA, USA; Center for Surgery and Public Health, Brigham and Women’s Hospital, Harvard Medical School, Boston, MA, USA; Center for Surgery and Public Health, Brigham and Women’s Hospital, Harvard Medical School, Boston, MA, USA; Division of Urological Surgery, Brigham and Women’s Hospital, Harvard Medical School, Boston, MA, USA; Center for Surgery and Public Health, Brigham and Women’s Hospital, Harvard Medical School, Boston, MA, USA; Center for Surgery and Public Health, Brigham and Women’s Hospital, Harvard Medical School, Boston, MA, USA; Division of Urological Surgery, Brigham and Women’s Hospital, Harvard Medical School, Boston, MA, USA; Center for Surgery and Public Health, Brigham and Women’s Hospital, Harvard Medical School, Boston, MA, USA; Section of Cancer Epidemiology and Health Outcomes, Rutgers Cancer Institute of New Jersey, New Brunswick, NJ, USA; Division of Urological Surgery, Brigham and Women’s Hospital, Harvard Medical School, Boston, MA, USA; Center for Surgery and Public Health, Brigham and Women’s Hospital, Harvard Medical School, Boston, MA, USA

## Abstract

**Background:**

The United States Preventive Services Task Force recommended against prostate-specific antigen (PSA) screening in 2012, which was modified in 2018 into shared decision making for men aged 55-70 years with a life expectancy over 10 years. We studied the trends in PSA screening in younger Black and White men with the implementation of the 2012 and 2018 guidelines.

**Methods:**

Younger Black and White men (aged 40-54 years) were identified using the Behavioral Risk Factor Surveillance System database biennially from 2012 to 2020. Our primary outcome was PSA screening within 2 years of the survey. An adjusted logistic regression model with 2-way interaction assessment between race and survey year was used to investigate the temporal trend of PSA screening in younger Black and White men.

**Results:**

A total of 142 892 men were included. We saw steadily decreasing odds of PSA screening among both younger Black and White men in 2014, 2016, 2018, and 2020 compared with 2012 (for younger Black men: odds ratio [OR]_2014_ = 0.77, 95% confidence interval [CI] = 0.62 to 0.96, OR_2016_ = 0.51, 95% CI = 0.41 to 0.63, OR_2018_ = 0.33, 95%CI = 0.27 to 0.42, OR_2020_ = 0.25, 95% CI = 0.18 to 0.32; and for younger White men: OR_2014_ = 0.81, 95% CI = 0.76 to 0.87, OR_2016_ = 0.66, 95% CI = 0.61 to 0.71, OR_2018_ = 0.41, 95%CI = 0.37 to 0.44, OR_2020_ = 0.36, 95% CI = 0.33 to 0.39). Younger Black men showed a brisker decrease in PSA screening in 2016, 2018, and 2020 compared with younger White men (all *P* < .05).

**Conclusions:**

PSA screening among younger men steadily decreased over the past decade since the 2012 United States Preventive Services Task Force guidelines, demonstrating a narrowing racial gap. How such an observed trend translates to long-term clinical outcomes for younger Black men remains to be seen.

Prostate cancer is the most common noncutaneous malignancy affecting men in the United States (1). The 2012 United States Preventive Task Force (USPSTF) guidelines recommended against routine screening for prostate cancer with prostate-specific antigen (PSA) (2). The guideline was subsequently modified in 2018 to advise shared decision making among men aged 55 to 70 years with a life expectancy of more than 10 years (3). Younger men (hereafter, referring to men between 40 and 54 years old) were consistently advised against routine screening with the hope of reducing overtreatment from overscreening in this low-risk group (2,3).

Black men experience higher prostate cancer incidence, earlier age of diagnosis, more advanced disease at diagnosis, poorer access to diagnostics and treatments, and ultimately, worse outcomes (4-11). Although the latest USPSTF guidelines recommended against screening in all younger men, PSA screening intensity and duration in younger Black men continues to be a subject of ongoing research and debate (12). Data from major clinical trials such as the Prostate, Lung, Colorectal, and Ovarian Cancer Screening Study; the European Randomized Study of Screening for Prostate Cancer; the Göteborg prostate cancer screening trial; and the Cluster Randomized Trial of PSA Testing for Prostate Cancer generated controversial conclusions (13-17). These major clinical trials on prostate cancer screening also included limited numbers of Black participants, raising the concern that their findings may not be generalizable to Black men (13-17). Modeling studies informed by natural history, age, and racial or ethnic differences within national databases have suggested that targeted PSA screening strategies that apply an earlier screening age for Black men could provide survival benefits that outweigh harms of overtreatment (18,19).

Prior research suggested a racial difference in PSA screening among younger men in the United States, with Black men being statistically significantly more likely to be screened with PSA at an earlier age (20,21). These studies were cross-sectional, and further investigations are needed to understand how such patterns have changed with the recent change of USPSTF prostate cancer screening guidelines in 2018. We aim to investigate the temporal trends of PSA screening in younger Black and White men over the past decade from 2012 and through the 2018 USPSTF guideline updates.

## Materials and methods

### Data source

We followed the Strengthening the Reporting of Observational Studies in Epidemiology Guidelines for cross-sectional studies in the reporting of this study. This study was approved by the institutional review board of Brigham and Women’s Hospital. Data were obtained from the Behavioral Risk Factor Surveillance System (BRFSS) database from 2012, 2014, 2016, 2018, and 2020. BRFSS is the largest longitudinal national survey system of the United States maintained by the Center for Disease Control and Prevention for health-related risk behaviors, chronic health conditions, and the use of preventive services. The database is based on annual surveys conducted via telephone calls to create a stratified random representative sample of adult residents in the United States. Patients were weighted by age, sex, race and ethnicity, educational level, marital status, property ownership, and telephone ownership. The median weighted response rates during the study period were 45.2%, 47.9%, 47.0%, 49.9%, and 47.9%, respectively, for each year of data included.

### Study population

We included non-Hispanic Black and White men between 40 and 54 years of age without prior history of prostate cancer who answered on BRFSS questionnaires whether they received a PSA test in the past 2 years. The variable “PSATIME” was used for selecting patients who received screening in the past 2 years. Patients with a history of prostate cancer or with missing data were excluded. The odd-numbered years between 2012 and 2020 were not included because questions regarding preventive health behaviors were only administered biennially.

### Outcome measures and covariates

Our primary outcome was the receipt of PSA screening within 2 years before the completion of the BRFSS survey. Our primary covariates of interest were race and ethnicity and year of BRFSS survey. The race and ethnicity were self-reported in the BRFSS database in the variable “RACE” and were categorized into Hispanic, non-Hispanic American Indian or Alaskan Native only, non-Hispanic Asian, non-Hispanic Black, non-Hispanic Native Hawaiian or other Pacific Islander, non-Hispanic Multiracial, non-Hispanic Other race, non-Hispanic White, and unsure/refused/do not know. Other covariates included were age at the time of survey, education level, annual income, insurance coverage, marital status, smoking status, body mass index, self-reported overall status of health, and having a personal doctor or not.

### Statistical analysis

We calculated the descriptive summary statistics for all variables across the included years of BRFSS data. Baseline characteristics of each covariate were compared via Pearson’s χ^2^ test accounting for the complex survey design. We performed a multivariable logistic regression accounting for complex survey design to estimate the adjusted odds ratio between PSA screening prevalence and the demographics in the national population. Covariates adjusted for in the model included age at the time of survey, education level, annual income, insurance coverage, marital status, smoking status, body mass index, self-reported overall status of health, and having a personal doctor or not. We performed a 2-way interaction assessment between race and ethnicity and year of BRFSS survey. This allowed us to analyze the marginal effects of White and Black younger men across different survey years on PSA screening via the logistic regression model with interaction term between survey year and race and ethnicity. We calculated the adjusted odds ratio of PSA screening between younger White and Black men throughout the years of BRFSS survey to compare and contrast their differences.

Two-sided statistical significance levels were set at *P* less than .05. Statistical analyses were performed using Stata version 16 (StataCorp LLC, College Station, TX, USA).

## Results

### Baseline demographics

A total of 142 892 men met the inclusion criteria. Among the included men from the 2012, 2014, 2016, 2018, and 2020 cohorts, a total of 28.5%, 25.6%, 22.1%, 15.5%, and 13.5%, respectively, received PSA screening in the past 2 years. Weighted and unweighted baseline characteristics of study cohort stratified by survey year were shown in Table 1.

**Table 1.** Baseline demographic characteristics of Non-Hispanic Black and White men aged 40-54 years in the behavioral risk factor surveillance system from 2012 to 2020^a^

	Total		2012		2014		2016		2018		2020	
	N = 142 892		N = 33 097		N = 30 424		N = 29 238		N = 26 583		N = 23 550	
	% (No.)	Est population in millions (95% CI)	% (No.)	Est population in millions (95% CI)	% (No.)	Est population in millions (95% CI)	% (No.)	Est population in millions (95% CI)	% (No.)	Est population in millions (95% CI)	% (No.)	Est population in millions (95% CI)
Age, y												
40-44	27.66 (39 519)	5.9 (5.8 to 6.0)	27.38 (9062)	1.4 (1.3 to 1.4)	26.90 (8185)	1.3 (1.2 to 1.3)	26.16 (7649)	1.2 (1.1 to 1.2)	27.81 (7394)	1.1 (1.1 to 1.2)	30.70 (7229)	1.0 (0.9 to 1.0)
45-49	32.06 (45 808)	5.4 (5.3 to 5.5)	32.05 (10 609)	1.3 (1.3 to 1.4)	31.28 (9517)	1.1 (1.1 to 1.2)	32.42 (9479)	1.2 (1.1 to 1.2)	32.67 (8685)	1.0 (1.0 to 1.0)	31.92 (7518)	0.8 (0.8 to 0.8)
50-54	40.29 (57 565)	6.9 (6.9 to 7.0)	40.57 (13 426)	1.6 (1.6 to 1.7)	41.82 (12 722)	1.5 (1.5 to 1.6)	41.42 (12 110)	1.5 (1.4 to 1.5)	39.51 (10 504)	1.3 (1.2 to 1.3)	37.38 (8803)	1.0 (1.0 to 1.1)
Having HCP												
No or unsure	21.42 (30 612)	4.0 (3.9 to 4.1)	19.54 (6466)	0.9 (0.9 to 1.0)	21.10 (6420)	0.9 (0.8 to 0.9)	20.66 (6040)	0.8 (0.7 to 0.8)	22.77 (6053)	0.8 (0.7 to 0.8)	23.92 (5633)	0.7 (0.6 to 0.7)
Has HCP	78.58 (112 280)	14 (14 to 14)	80.46 (26 631)	3.4 (3.3 to 3.4)	78.90 (24 004)	3.1 (3.0 to 3.1)	79.34 (23 198)	3.0 (2.9 to 3.0)	77.23 (20 530)	2.6 (2.5 to 2.7)	76.08 (17 917)	2.1 (2.1 to 2.2)
Health insurance												
No or unsure	10.76 (15 370)	2.2 (2.2 to 2.3)	13.82 (4574)	0.7 (0.7 to 0.7)	10.01 (3045)	0.5 (0.4 to 0.5)	9.17 (2681)	0.4 (0.4 to 0.4)	10.14 (2696)	0.4 (0.4 to 0.4)	10.08 (2374)	0.3 (0.3 to 0.3)
Yes	89.24 (127 522)	16 (16 to 16)	86.18 (28 523)	3.6 (3.6 to 3.6)	89.99 (27 379)	3.5 (3.4 to 3.5)	90.83 (26 557)	3.4 (3.3 to 3.4)	89.86 (23 887)	3.0 (2.9 to 3.1)	89.92 (21 176)	2.5 (2.5 to 2.6)
Health status												
Excellent	19.92 (28 471)	3.6 (3.5 to 3.6)	19.79 (6550)	0.8 (0.8 to 0.9)	19.68 (5987)	0.8 (0.7 to 0.8)	18.58 (5433)	0.7 (0.7 to 0.7)	18.49 (4914)	0.6 (0.6 to 0.7)	23.72 (5587)	0.7 (0.6 to 0.7)
Very good	37.22 (53 185)	6.5 (6.4 to 6.6)	36.95 (12 230)	1.5 (1.5 to 1.6)	37.71 (11 473)	1.4 (1.3 to 1.4)	36.67 (10 722)	1.3 (1.3 to 1.4)	36.07 (9588)	1.1 (1.1 to 1.2)	38.95 (9172)	1.1 (1.0 to 1.1)
Good	29.75 (42 505)	5.5 (5.4 to 5.5)	29.20 (9664)	1.3 (1.2 to 1.3)	29.73 (9045)	1.2 (1.1 to 1.2)	30.95 (9048)	1.2 (1.1 to 1.2)	31.06 (8258)	1.1 (1.0 to 1.1)	27.56 (6490)	0.8 (0.7 to 0.8)
Fair	9.49 (13 559)	1.9 (1.8 to 1.9)	10.02 (3316)	0.5 (0.5 to 0.5)	9.21 (2803)	0.4 (0.4 to 0.4)	10.06 (2940)	0.4 (0.4 to 0.4)	10.39 (2763)	0.4 (0.3 to 0.4)	7.38 (1737)	0.2 (0.2 to 0.2)
Poor	3.62 (5172)	0.7 (0.7 to 0.8)	4.04 (1337)	0.2 (0.2 to 0.2)	3.67 (1116)	0.2 (0.2 to 0.2)	3.75 (1095)	0.2 (0.1 to 0.2)	3.99 (1060)	0.1 (0.1 to 0.2)	2.39 (564)	0.7 (0.6 to 0.8)
Income												
<$15 000	6.53 (9337)	1.3 (1.2 to 1.3)	8.00 (2647)	0.4 (0.4 to 0.4)	6.99 (2126)	0.3 (0.3 to 0.3)	6.49 (1897)	0.3 (0.2 to 0.3)	5.89 (1565)	0.2 (0.2 to 0.2)	4.68 (1102)	0.1 (0.1 to 0.1)
$15 000 to <$25 000	9.07 (12 960)	1.8 (1.7 to 1.8)	9.96 (3298)	0.5 (0.4 to 0.5)	9.23 (2808)	0.4 (0.4 to 0.4)	9.24 (2703)	0.4 (0.3 to 0.4)	8.91 (2368)	0.3 (0.3 to 0.3)	7.57 (1783)	0.2 (0.2 to 0.3)
$25 000 to <$35 000	6.23 (8907)	1.1 (1.1 to 1.2)	7.13 (2360)	0.3 (0.3 to 0.3)	6.29 (1913)	0.3 (0.2 to 0.3)	6.37 (1863)	0.3 (0.2 to 0.3)	5.83 (1549)	0.2 (0.2 to 0.2)	5.19 (1222)	0.1 (0.1 to 0.2)
$35 000 to <$50 000	11.06 (15 806)	2.0 (1.9 to 2.0)	12.53 (4148)	0.6 (0.5 to 0.6)	11.66 (3546)	0.4 (0.4 to 0.5)	11.26 (3291)	0.4 (0.4 to 0.4)	9.94 (2642)	0.3 (0.3 to 0.3)	9.25 (2179)	0.3 (0.2 to 0.3)
≥$50 000	67.10 (95 882)	1.2 (1.2 to 1.2)	62.37 (20 644)	0.3 (0.3 to 0.3)	65.84 (20 031)	0.3 (0.2 to 0.3)	66.64 (19 484)	0.3 (0.2 to 0.3)	69.44 (18 459)	0.2 (0.2 to 0.2)	73.31 (17 264)	0.2 (0.2 to 0.2)
Level of education												
Less than high school	4.94 (7065)	1.6 (1.6 to 1.7)	5.37 (1776)	0.4 (0.4 to 0.5)	4.80 (1459)	0.3 (0.3 to 0.4)	5.19 (1516)	0.3 (0.3 to 0.4)	4.84 (1287)	0.3 (0.3 to 0.3)	4.36 (1027)	0.2 (0.2 to 0.3)
High school	27.66 (39 519)	5.4 (5.4 to 5.5)	29.40 (9732)	1.4 (1.3 to 1.4)	27.62 (8404)	1.2 (1.2 to 1.2)	28.06 (8205)	1.1 (1.1 to 1.2)	26.68 (7092)	0.9 (0.9 to 1.0)	25.84 (6086)	0.8 (0.7 to 0.8)
College/tech	25.77 (36 822)	5.5 (5.4 to 5.5)	25.52 (8446)	1.2 (1.2 to 1.3)	25.32 (7702)	1.2 (1.1 to 1.2)	25.72 (7519)	1.1 (1.1 to 1.2)	26.36 (7008)	1.0 (9.8 to 1.1)	26.10 (6147)	0.8 (0.8 to 0.9)
Graduated college/tech	41.63 (59 486)	5.8 (5.7 to 5.8)	39.71 (13 143)	1.3 (1.3 to 1.3)	42.27 (12 859)	1.2 (1.2 to 1.2)	41.04 (11 998)	1.2 (1.1 to 1.2)	42.12 (11 196)	1.1 (1.1 to 1.1)	43.69 (10 290)	1.0 (0.9 to 1.0)
Marital status												
Never married	13.44 (19 206)	2.6 (2.5 to 2.7)	13.98 (4626)	0.7 (0.7 to 0.7)	13.28 (4040)	0.5 (0.5 to 0.6)	13.59 (3972)	0.5 (0.5 to 0.6)	13.31 (3539)	0.4 (0.4 to 0.5)	12.86 (3029)	0.4 (0.3 to 0.4)
Married	67.56 (96 543)	12 (12 to 12)	66.50 (22 010)	2.8 (2.8 to 2.9)	68.26 (20 767)	2.6 (2.6 to 2.7)	67.13 (19 628)	2.5 (2.4 to 2.5)	66.94 (17 795)	2.3 (2.2 to 2.3)	69.40 (16 343)	1.9 (1.9 to 2.0)
Other	19.00 (27 143)	3.3 (3.3 to 3.4)	19.52 (6461)	0.8 (0.8 to 0.8)	18.46 (5617)	0.7 (0.7 to 0.7)	19.28 (5638)	0.7 (0.7 to 0.8)	19.75 (5249)	0.6 (0.6 to 0.6)	17.74 (4178)	0.5 (0.5 to 5.0)
Race and ethnicity												
Black	9.16 (13 085)	2.8 (2.8 to 2.9)	9.09 (3007)	0.7 (0.6 to 0.7)	8.39 (2552)	6.0 (5.6 to 6.4)	9.39 (2745)	0.6 (0.6 to 0.6)	10.09 (2681)	0.6 (0.5 to 0.6)	8.92 (2100)	0.5 (0.4 to 0.5)
White	90.84 (129 807)	15 (15 to 15)	90.91 (30 090)	3.6 (3.6 to 3.7)	91.61 (27 872)	3.4 (3.3 to 3.4)	90.61 (26 493)	3.3 (3.3 to 3.4)	89.91 (23 902)	2.8 (2.7 to 2.8)	91.08 (21 450)	2.3 (2.3 to 2.4)
Received PSA screening in last 2 y												
No	78.26 (111 827)	14 (14 to 14)	71.11 (23 535)	3.1 (3.1 to 3.2)	74.73 (22 735)	2.9 (2.9 to 3.0)	77.57 (22 679)	2.9 (2.9 to 3.0)	84.46 (22 451)	2.8 (2.8 to 2.9)	86.74 (20 427)	2.4 (2.3 to 2.5)
Yes	21.74 (31 065)	3.9 (3.9 to 4.0)	28.89 (9562)	1.2 (1.2 to 1.3)	25.27 (7689)	1.0 (1.0 to 1.0)	22.43 (6559)	0.8 (0.8 to 0.9)	15.54 (4132)	0.5 (0.5 to 0.5)	13.26 (3123)	3.8 (3.5 to 4.0)
Smoking status												
Never smoker	55.70 (79 585)	9.6 (9.5 to 9.7)	54.97 (18 194)	2.2 (2.2 to 2.3)	57.47 (17 486)	2.1 (2.1 to 2.2)	55.59 (16 253)	1.9 (1.9 to 2.0)	55.60 (14 781)	1.8 (1.7 to 1.8)	54.65 (12 871)	1.5 (1.4 to 1.5)
Former smoker	24.91 (35 592)	4.6 (4.5 to 4.7)	24.54 (8122)	1.1 (1.0 to 1.1)	23.63 (7188)	1.0 (0.9 to 1.0)	24.93 (7288)	1.0 (0.9 to 1.0)	25.32 (6730)	0.9 (0.8 to 0.9)	26.60 (6264)	0.7 (0.7 to 0.8)
Current smoker	19.40 (27 715)	3.9 (3.8 to 4.0)	20.49 (6781)	1.0 (1.0 to 1.0)	18.90 (5750)	0.8 (0.8 to 0.9)	19.48 (5697)	0.8 (0.8 to 0.9)	19.08 (5072)	0.7 (0.6 to 0.7)	18.75 (4415)	0.6 (0.5 to 0.6)
Weight status, BMI												
<25	20.34 (29 070)	3.6 (3.5 to 3.7)	21.42 (7091)	0.9 (0.8 to 0.9)	21.28 (6474)	0.7 (0.7 to 0.7)	20.10 (5876)	0.7 (0.7 to 0.8)	19.36 (5146)	0.6 (0.6 to 0.7)	19.04 (4483)	0.5 (0.5 to 0.6)
25-30	42.81 (61 179)	7.7 (7.6 to 7.8)	44.89 (14 858)	1.9 (1.9 to 2.0)	43.58 (13 258)	1.7 (1.6 to 1.7)	42.48 (12 419)	1.6 (1.6 to 1.6)	41.29 (10 976)	1.4 (1.3 to 1.4)	41.05 (9668)	1.1 (1.1 to 1.2)
>30	36.84 (52 643)	6.7 (6.6 to 6.8)	33.68 (11 148)	1.5 (1.4 to 1.5)	35.14 (10 692)	1.4 (1.4 to 1.5)	37.43 (10 943)	1.4 (1.4 to 1.5)	39.35 (10 461)	1.3 (1.3 to 1.3)	39.91 (9399)	1.1 (1.1 to 1.2)

a BMI = body mass index; CI = confidence interval; HCP = health-care provider; PSA = prostate-specific antigen.

### Multivariable logistic regression and interaction analysis

**Figure 1.**
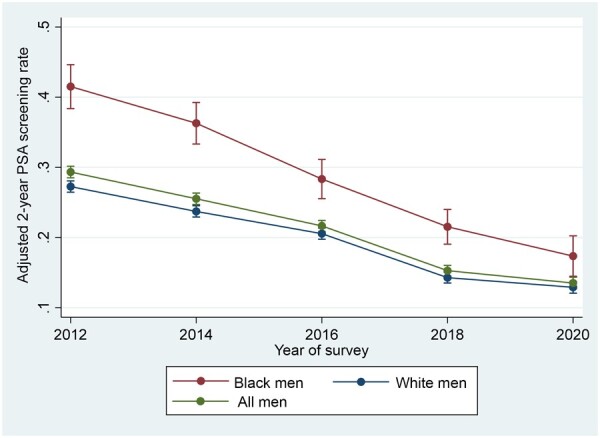
Adjusted prostate-specific antigen screening rates in younger Black and White men aged 40 to 54 years from 2012 to 2020.

In our multivariable model, we found that both primary covariates of interest, survey year and race, were statistically significantly associated with receiving a PSA test within 2 years before survey completion (Table 2). Analysis of marginal effects of interaction between race and survey year adjusting for the above-mentioned clinical and demographic covariates allowed us to estimate the PSA screening rates during the study period under the counterfactual scenarios where the total population was entirely Black or White (Figure 1). The adjusted 2-year PSA screening rates for White men were 27.1%, 23.6%, 20.5%, 14.4%, and 12.9%, respectively, for years 2012 to 2020. In parallel, the adjusted 2-year PSA screening rates for Black men were 41.4%, 36.7%, 28.2%, 21.4%, and 17.5%, respectively, for years 2012 to 2020 (Figure 1). The adjusted odds ratios of receiving PSA screening among both younger White and Black men were statistically significantly lower in 2014, 2016, 2018, and 2020 in reference to 2012 (for younger Black men: odds ratio [OR]_2014_ = 0.77, 95% confidence interval [CI] = 0.62 to 0.96, OR_2016_ = 0.51, 95% CI = 0.41 to 0.63, OR_2018_ = 0.33, 95% CI = 0.27 to 0.42, OR_2020_ = 0.25, 95% CI = 0.18 to 0.32; and for younger White men: OR_2014_ = 0.81, 95% CI = 0.76 to 0.87, OR_2016_ = 0.66, 95% CI = 0.61 to 0.71, OR_2018_ = 0.41, 95% CI = 0.37 to 0.44, OR_2020_ = 0.36, 95% CI = 0.33 to 0.39) (Table 3). Younger Black men showed a more rapid decrease in PSA screening compared with younger White men in years 2016, 2018, and 2020 (*P* = .02, .02, .01). Such statistical significance was not observed in year 2014 (*P* = .68) (Table 3). The adjusted odds ratios between younger Black and White men showed consistently higher PSA screening among Black men throughout survey years (Table 4).

**Table 2.** Adjusted logistic regression with patient-level predictors of prostate-specific antigen screening^a^

	Odds ratio (95% CI)
Age, y	
40-44	Ref
45-49	1.88 (1.75 to 2.02)
50-54	4.44 (4.15 to 4.74)
Education	
Less than high school	Ref
Graduated high school	1.44 (1.25 to 1.67)
College/tech	1.69 (1.46 to 1.96)
Graduated college/tech	1.87 (1.61 to 2.17)
Having health-care provider	
No or unsure	Ref
Yes	2.95 (2.72 to 3.19)
Health coverage	
No or unsure	Ref
Yes	1.85 (1.66 to 2.05)
Health status	
Excellent	Ref
Very good	0.97 (0.90 to 1.04)
Good	0.90 (0.84 to 0.98)
Fair	0.93 (0.84 to 1.04)
Poor	1.10 (0.94 to 1.28)
Income	
<$15 000	Ref
$15 000 to <$25 000	1.15 (0.99 to 1.33)
$25 000 to <$35 000	1.15 (0.98 to 1.34)
$35 000 to <$50 000	1.24 (1.07 to 1.43)
≥$50 000	1.52 (1.32 to 1.74)
Married	
Never married	Ref
Married/couple	1.26 (1.16 to 1.38)
Other/divorced/widowed/separated	1.22 (1.11, 1.34)
Race	
White	Ref
Black	2.11 (1.79-2.47)
Smoking status	
Never smoker	Ref
Former smoker	1.04 (0.98 to 1.10)
Current smoker	0.85 (0.79 to 0.92)
Weight status, BMI	
<25	Ref
25-30	1.05 (0.98, 1.13)
>30	1.13 (1.06, 1.22)
Year	
2012	Ref
2014	0.81 (0.76 to 0.87)
2016	0.66 (0.61 to 0.71)
2018	0.41 (0.37 to 0.44)
2020	0.36 (0.33 to 0.39)

a Interaction terms and marginal analyses were reported separately in Tables 3 and 4. BMI = body mass index; CI = confidence interval.

**Table 3.** Adjusted odds ratios for prostate-specific antigen screening between younger Black and White men aged 40 to 54 years from 2012 to 2020^a^

Year	Odds ratios for younger Black men (95% CI)	Odds ratios for younger White men (95% CI)	*P* between younger Black and White Men	Pooled odds ratios with 2012 as reference (95% CI)
2012	Ref	Ref		Ref
2014	0.77 (0.62 to 0.96)	0.81 (0.76 to 0.87)	.680	0.80 (0.75 to 0.86)
2016	0.51 (0.41 to 0.63)	0.66 (0.61 to 0.71)	.024	0.63 (0.59 to 0.68)
2018	0.33 (0.27 to 0.42)	0.41 (0.37 to 0.44)	.018	0.40 (0.36 to 0.42)
2020	0.25 (0.18 to 0.32)	0.36 (0.33 to 0.39)	.012	0.33 (0.31 to 0.37)

a CI = confidence interval.

**Table 4.** Adjusted odds ratios for prostate-specific antigen screening in younger Black and White men aged 40 to 54 years within each year^a^

Year	Odds ratios for younger Black vs White men (95% CI)
2012	2.11 (1.79 to 2.47)
2014	2.01 (1.71 to 2.35)
2016	1.61 (1.37 to 1.91)
2018	1.73 (1.45 to 2.07)
2020	1.46 (1.15 to 1.85)
Pooled younger Black vs White men	1.83 (1.69 to 1.98)

a CI = confidence interval.

Additional covariates that showed statistically significant associations with PSA screening included age group, marital status, income group, education, body mass index, smoking status, health insurance coverage, and having a personal doctor (Table 2). A more advanced age group was associated with higher PSA screening (OR = 1.88, 95% CI = 1.75 to 2.02 for 45-50 years; 4.44, 95% CI = 4.15 to 4.74 for >50 years). Younger men with a BMI greater than 30 showed 1.13 times the odds of PSA screening (OR = 1.13, 95% CI = 1.06 to 1.22). Higher income was associated with greater odds of PSA screening (OR = 1.24, 95% CI = 1.07 to 1.43 for $35 000-$50 000; 1.52, 95% CI = 1.32 to 1.74 for ≥$50 000). Having health insurance coverage was associated with 1.85 times the odds of screening (OR = 1.85, 95% CI = 1.66 to 2.05). Having a health-care provider was linked with a 2.95 times increased odds of PSA screening (OR = 2.95, 95% CI = 2.72 to 3.19).

## Discussion

Using national data from BRFSS over the past decade, we observed decreasing PSA screening among younger Black and White men between 40 and 54 years of age. While our results of higher rates of PSA screening in younger Black men concurred with the existing literature, we found that younger Black men exhibited a more rapid decline compared with their White counterparts, resulting in a narrowing racial gap.

Our PSA screening rates among younger men were consistent with earlier reports using BRFSS data (20,21). Our results from year 2012 aligned with the racial gap of higher PSA screening rate among younger Black men observed by Sammon et al. (20). We also noticed a similar overall decrease in screening across different races since the 2012 guideline as reported by Kensler et al. (21). Secondary analysis from the same study also reported a more rapid decrease in PSA screening among Black men from 2012 to 2018, especially those of a younger age (21). We also saw evidence of these patterns in our pooled multivariable analysis, which included additional years of follow-up (Table 2).

Evidence suggesting higher risk of prostate cancer in Black men along with insufficient data to make a recommendation regarding PSA screening from earlier 2008 guidelines likely together contributed to a higher screening rate among Black men in 2012. Although the effects of the 2018 USPSTF guideline change are still underway, the 2012 USPSTF guideline recommendations against routine PSA screening is the most likely explanation for the overall decrease in screening rate within young men in our study population (2,3). From the provider perspective, USPSTF guidelines had profound impacts on daily clinical practices, translating to less physician initiation of PSA screening (22,23). From a health systems perspective, the recommendation against PSA use made insurance reimbursement more challenging for patients tested for PSA. With the rapid diffusion of the Accountable Care Organization model from Medicare that emphasizes the value-based care by abiding by established guidelines, data suggested that PSA screening decreased as a result (24).

Although less screening would reduce harms of overdiagnosis and overtreatment, there is still debate about whether mortality benefits may accrue to certain high-risk subgroups (25). Jemal et al. (22) highlighted the consequence of fewer prostate cancer diagnoses after the 2012 guideline changes. More than 33 000 cases of prostate cancer could be missed as a result of the 2012 guideline change, which correlates with the overall decreasing trend of prostate cancer incidence in the last decade (22). While a significant proportion of these now not-diagnosed prostate cancers were likely previously overdiagnosed, some could be clinically significant prostate cancer. Recent data suggested an increasing incidence of more advanced prostate cancer at diagnosis since the USPSTF guideline changes in 2012 (26,27). Desai et al. (27) analyzed data from the Surveillance, Epidemiology, and End Results (registry from 2004 to 2018 and found increasing incidence since the 2012 guideline changes in men younger than 75 years. Jemal et al. (22) similarly found increasing regional or distant metastatic prostate cancer between 2012 and 2016 among men aged 50-74 years. This trend was observed in both Black and White men, with Black men being 2-3 times more likely to have distant disease. Combined with our results, these findings raise the question of whether the observed decrease in screening among younger men might have contributed to such an increase, potentially having a greater effect on younger Black men.

Prostate cancer in younger Black men is an understudied clinical entity. More than 10% of all prostate cancers are diagnosed in men younger than 55 years of age, a subgroup of which was more aggressive early-onset disease that led to worse overall survival in young men compared with those diagnosed with prostate cancer at more advanced ages (28). Many prior reports showed a steadily increasing incidence of prostate cancer in men younger than 55 years over the past decade as a result of both lifestyle factors and screening (28,29). However, clinical trials studying PSA screening and best modality for prostate cancer treatment often failed to include sufficient numbers of younger Black men to optimize screening strategies in these groups. Studies in the past proposed the idea of using a base-line midlife PSA after age 40 years to help guide further screening (25,30,31). Younger men with markedly elevated PSA compared with age-adjusted medians had statistically significantly increased risk of subsequent development of aggressive prostate cancer (25,30,32). Parallel results were also observed in a cohort of Black men aged 40 to 54 years, suggesting the possible value of a baseline PSA that could inform future USPSTF recommendations (25). Our study added to the limited literature describing the preventive health behaviors in this patient population and hopefully could pave ways for future studies that may help correlate screening with long-term clinical outcomes in younger men.

The results of this study should be interpreted within its limitations. Firstly, the data of this study were based on a national survey. The self-reported PSA screening rate was subject to inherent recall bias and could not be validated by review of individual medical records. The telephone survey–based nature of BRFSS also inherently conferred sampling bias, because adults with limited access to phone, unwilling to participate in the lengthy survey, and those with language barriers were less likely to be included. The response rates of BRFSS were approximately 50% during the study period; however, our analyses were weighted to mitigate the effect of nonresponse. BRFSS also did not capture data regarding prostate cancer mortality, precluding us from further elucidating the clinical outcomes of changes in PSA screening. However, the strength of our study stemmed from our use of the largest continuous health survey maintained by the Centers for Disease Control and Prevention, leading to a demographically diverse population with robust statistical power to compare the odds of nonrecommended PSA screening in different races and ethnicities among younger men of different age groups. By performing unadjusted descriptive statistics, multivariable regression, interaction analysis, and average marginal effect analysis in sequential order, we were able to compare the trend and velocity of change at each available time point with decent clarity.

PSA screening among younger men aged 40-54 years steadily decreased over the past decade since the 2012 USPSTF guideline change, leading to a closing gap between younger White and Black men. How such observed trends translate to long-term clinical outcomes remains to be seen.

## Data Availability

Data underlying this article is available at https://www.cdc.gov/brfss/annual_data/annual_data.htm.

## Notes

**Role of the funder:** Not applicable.

**Disclosures:** QDT reports personal fees from Astellas, Bayer, and Janssen, outside the submitted work. QDT reports research funding from the American Cancer Society, the Defense Health Agency, and Pfizer Global Medical Grants. APC reports research funding from the American Cancer Society and Pfizer Global Medical Grants.

**Author contributions:** Conceptualization (ZQ, SL, APC, HSI, QDT), Formal Analysis (ZQ, KAK, XC) Software (ZQ, KAK, XC), Visualization (SB), Writing—Original Draft (ZQ, KAK), Writing—review and editing (ZQ, KAK, XC, ML, SL, APC, QDT), Supervision (QDT).
